# Growth, puberty, and bone health in children and adolescents with inflammatory bowel disease

**DOI:** 10.1186/s12887-021-02496-4

**Published:** 2021-01-14

**Authors:** Hye-Young Jin, Jae-Sang Lim, Yena Lee, Yunha Choi, Seak-Hee Oh, Kyung-Mo Kim, Han-Wook Yoo, Jin-Ho Choi

**Affiliations:** 1 Department of Pediatrics, Center for Pediatric Cancer, National Cancer Center, Goyang, Gyeonggi-do Republic of Korea; 2 Department of Pediatrics, Asan Medical Center, University of Ulsan College of Medicine, 88, Olympic-Ro 43-Gil, Songpa-Gu, 05505 Seoul, Republic of Korea

**Keywords:** Growth, Puberty, Bone mineral density, Inflammatory bowel disease, Crohn disease, Ulcerative colitis

## Abstract

**Background:**

Endocrine complications such as impaired growth, delayed puberty, and low bone mineral density (BMD) can be associated with inflammatory bowel disease (IBD) in children and adolescents. This study was performed to investigate the frequency, characteristics, and outcomes of endocrine complications of IBD in children and adolescents.

**Methods:**

This study included 127 patients with IBD diagnosed before 18 years of age [117 with Crohn disease (CD) and 10 with ulcerative colitis (UC)]. Growth profiles, pubertal status, 25-hydroxyvitamin D_3_ [25(OH)D_3_] levels, and BMD were reviewed retrospectively.

**Results:**

Short stature was observed in 14 of 127 (11.0 %) with a mean height-SDS of -2.31 ± 0.72. During a 2-year follow-up period, height-SDS did not significantly improve, while weight-SDS significantly improved. Among 109 patients who were older than 13 (girls) or 14 (boys) years of age during the study period, 11 patients (10.1 %) showed delayed puberty, which was associated with low weight-SDS. Vitamin D deficiency was documented in 81.7 % (94/115) with the average 25(OH)D_3_ level of 14.5 ± 7.0 ng/mL. Lumbar BMD Z-score was below − 2 SDS in 25 of 119 patients (21.0 %). Height-SDS, weight-SDS, and body mass index (BMI)-SDS were lower in patients with osteoporosis than those without osteoporosis. When pediatric CD activity index scores were high (≥ 30), weight-SDS, BMI-SDS, insulin-like growth factor 1 (IGF-1)-SDS, and testosterone levels were significantly decreased.

**Conclusions:**

Vitamin D deficiency and osteoporosis are common in pediatric IBD patients. As disease severity deteriorates, weight-SDS, IGF-1-SDS, and testosterone levels were decreased. Optimal pubertal development is necessary for bone health.

## Background

Inflammatory bowel disease (IBD) is a chronic relapsing-remitting inflammatory condition divided into two phenotypes: Crohn disease (CD) and ulcerative colitis (UC) [1]. The global incidence and prevalence of IBD has been increasing worldwide [2]. The incidence and prevalence of IBD in Asia remains low compared with the incidence and prevalence seen in Western countries; however, this gap has been narrowing in recent years [3]. The mean prevalence of IBD in the total population of Western countries is estimated at 1/1000, while the prevalence in Korea has been reported to be 29.6/100,000 in CD and 66.0/100,000 in UC [4–6]. Among patients with IBD, approximately 25 % of patients manifest symptoms before the age of 18 years [7]. The highest reported annual pediatric incidence of IBD is 23 cases per 100,000 person-years in Europe and 11.4 cases per 100,000 person-years in Asia [8]. In Korea, recently published local data shows that incidence of pediatric IBD increased from 0.86 to 100,000 in 2011 to 3.33 per 100,000 in 2016 [9].

Previous studies have investigated the frequency and clinical characteristics of endocrine complications in children and adolescents with IBD, such as impaired growth, delayed puberty, and low bone mineral density (BMD) [10–16]. Growth profiles can help clinicians diagnose IBD in children and adolescents because growth retardation is commonly noted at diagnosis, and decreased height velocity has been reported to be the first clinical sign of IBD in 46 % of patients [7, 17]. Approximately 10–33 % of pediatric IBD patients manifested short stature (height < 3rd percentile) at diagnosis [18]. Previous reports demonstrated that 25–30 % of children with IBD had a deficit in final height [19, 20]. Although pediatric CD patients have normal growth hormone (GH) concentrations, low serum insulin-like growth factor-1 (IGF-1) levels caused by malnutrition and elevated proinflammatory cytokine levels contribute to growth impairment [11, 21]. Cytokines may interfere with GH and IGF-1 action and have direct effects on growth plates, rendering the growth plates less sensitive to IGF-1 [22]. Delayed puberty is also frequently found in patients with IBD [10, 18]. It is presumed that puberty is delayed due to abnormalities of sex steroid production or action caused by proinflammatory cytokines [10, 23]. Pro-inflammatory cytokines may inhibit the generation of sex hormones by acting on the gonads or by suppressing gonadotropin-releasing hormone (GnRH) [24, 25]. The timing of breast development, testicular enlargement, growth spurt, and menarche are also delayed in patients with IBD [18, 26, 27]. In addition, about 41.4–46.7 % of children and adolescents with IBD showed low BMD with a Z-score ≤ -1.0 [13, 28]. Risk factors of reduced BMD include glucocorticoid treatment, nutritional deficiencies of vitamin D and/or calcium, hypogonadism, and disease-related chronic inflammation [29].

As the number of children and adolescents diagnosed with IBD has gradually increased, careful documentation of endocrine complications in children and adolescents with IBD is critical for management. A comprehensive understanding of endocrine complications and multidisciplinary approach to IBD patients make it possible to provide better treatment and clinical outcome as well as management of gastrointestinal symptoms. To date, a few studies have focused on the analysis of endocrine complications of pediatric IBD in a limited number of cases [16, 30–32]. However, the pathogenesis, frequency, and outcomes of endocrine complications of IBD have not been fully established. Thus, this study was performed to investigate the frequency, clinical characteristics, and outcomes of endocrine complications in children and adolescents with IBD.

## Methods/design

### Subjects

This study retrospectively reviewed medical records of 127 patients who were diagnosed with IBD before 18 years of age and visited pediatric endocrinologic outpatient clinic between January 2005 and December 2017. Among the 127 patients, 117 patients were diagnosed with CD, and 10 patients were diagnosed with UC. All patients were followed up for at least two years. Diagnosis of IBD was based on clinical, radiologic, endoscopic, and histologic findings [33, 34]. Disease activity indices of CD using the pediatric CD activity index (PCDAI) were calculated by evaluating categories such as history items, physical examination items, and laboratory tests. Items were scored and PCDAI is the sum of all scores of each category (range: 0–100) [35]. Patients with CD were categorized into two groups according to PCDAI scoring: < 30 as inactive/mild and ≥ 30 as moderate/severe CD [36]. This study was approved by the Institutional Review Board of the Asan Medical Center, Seoul, Korea (IRB No. 2017 − 0939). Our study was exempt from the requirement of informed consent because of the retrospective nature of the study and the analysis used anonymous clinical data.

### Endocrinologic evaluation

Growth parameters including height, weight, body mass index (BMI, kg/m^2^), serum IGF-1, and IGF binding protein-3 (IGFBP-3), were reviewed. Height, weight, and BMI were expressed using standard deviation scores (SDSs) based on age- and sex-matched normative data from Korean references [37]. Serum IGF-1 and IGFBP-3 levels were measured using an immunoradiometric assay (IRMA, Immunotech, Marseilles, France) and were expressed as a SDS based on age- and sex-matched normative data from Korean references [38]. Puberty was assessed by the following parameters: Tanner stage; bone age (BA); and serum luteinizing hormone (LH), follicle stimulating hormone (FSH), estradiol (in females), and testosterone levels (in males). The LH and FSH levels were determined by immunoradiometric assay (IRMAmat, Byk-Sangtec Diagnostica, Hessen, Germany). The estradiol and testosterone levels were measured by radioimmunoassay (Coat-A-Count, Diagnostic Products, Los Angeles, CA, USA). BA was determined by the Greulich-Pyle method [39]. BMD of the lumbar spine (L2–4) was measured by dual energy X-ray absorptiometry (DXA, Lunar Corp., Madison, WI, USA). BMD Z-score was adjusted using age- and sex-matched references for Korean children [40, 41]. Since Korean reference data was measured by the Hologic system, BMD data was converted using following formula: Hologic lumbar spine BMD = 0.837 × GE-Lunar lumbar spine BMD + 0.021 [42].

Short stature was defined as a height-SDS of less than − 2.0 according to age- and sex-matched Korean references [37]. Delayed puberty was defined as the absence of secondary sexual characteristics at age 14 years for males and 13 years for females [43]. Serum thyroid stimulating hormone (TSH) level was measured using an immunoradiometric assay (TSH-CTK-3®, DiaSorin, Saluggia, Italy). Serum free T4 level was determined by a radioimmunoassay (FT4 RIA Kit®, Beckman Coulter, Prague, Czech Republic). Vitamin D deficiency was defined as a 25(OH)D_3_ level less than 20 ng/mL [44]. The 25(OH)D_3_ level was measured using radioimmunoassay (Nichols Institute Diagnostics, San Clemente, CA, USA). Osteoporosis was defined as a BMD Z-score less than − 2.0 by measuring lumbar spine 2 to 4 [45].

### Statistical analyses

Statistical analyses were performed using IBM SPSS Statistics for Windows version 21.0 (IBM Corp., Armonk, NY, USA). To evaluate endocrine complications between two groups categorized by PCDAI scores, continuous variables such as anthropometric and biochemical data were analyzed by independent *t*-tests, while discrete variables such as presence of short stature, delayed puberty, vitamin D deficiency, and osteoporosis were analyzed by Fisher’s exact test. Mann-Whitney *U* test was used to compare anthropometric data between two groups classified according to the presence of delayed puberty because only 11 patients manifested delayed puberty. The independent *t* test was used to compare anthropometric and biochemical data of groups categorized according to the presence of osteoporosis. Pearson correlation analysis was used to assess the correlation between serum 25(OH)D_3_ level and lumbar spine BMD Z-score. *P* values < 0.05 were considered statistically significant.

## Results

### Growth impairment in patients with IBD

Age at time of diagnosis of included patients with IBD was 13.5 ± 2.5 years (range, 1.3–17.8 years). Initial height-SDS and weight-SDS were − 0.39 ± 1.06 and − 1.19 ± 1.38, respectively. Initial BMI-SDS was − 1.26 ± 1.40. Demographic data and clinical characteristics of the patients are described in Table 1.

**Table 1:** Demographic and clinical characteristics of the patients at diagnosis

	CD (*n* = 117)	UC (*n* = 10)	Total (*n* = 127)
Sex (male/female)	79/38	5/5	84/43
Age at diagnosis, years	13.5 ± 2.3 (2.0–17.8)	12.8 ± 4.5 (1.3–16.6)	13.5 ± 2.5 (1.3–17.8)
Height-SDS	-0.42 ± 1.08	-0.01 ± 0.83	-0.39 ± 1.06
Weight-SDS	-1.23 ± 1.39	-0.55 ± 1.14	-1.19 ± 1.38
BMI-SDS	-1.30 ± 1.41	-0.67 ± 1.22	-1.26 ± 1.40
Short stature	13/117	1/10	14/127 (11.0 %)
Delayed puberty	11/101	0/8	11/109 (10.1 %)
Vitamin D deficiency	87/106	7/9	94/115 (81.7 %)
25(OH)D_3_, ng/mL	14.55 ± 7.04 (2.2–45.2)	13.69 ± 5.85 (7.1–22.1)	14.49 ± 6.94 (2.2–45.2)
Osteoporosis	21/111	4/8	25/119 (21.0 %)
Lumbar spine BMD-SDS	-0.82 ± 1.29 (-4.07–4.36)	-0.61 ± 1.53 (-2.11–2.16)	-0.80 ± 1.31 (-4.07–4.36)

*CD *Crohn disease, *UC *Ulcerative colitis, *BMI *body mass index, *25(OH)D*_*3*_ 25-hydroxyvitamin D_3_, *BMD *Bone mineral density

Short stature was observed in 14 of 127 patients (11.0 %) including 13 patients with CD and one patient with UC. The average age at time of diagnosis of IBD in patients with short stature was 11.9 ± 3.6 years, and the mean age at time of IBD diagnosis in patients without short stature was 13.7 ± 2.3 years. The mean height-SDS and weight-SDS in 14 patients with short stature at time of diagnosis were − 2.31 ± 0.72 and − 2.34 ± 1.47, respectively. The BMI-SDS in the 14 patients with short stature was − 1.50 ± 1.46, which was not significantly different from the mean BMI (-1.22 ± 1.40) of the patients without short stature (*P* = 0.415). The BA/chronological age (CA) ratio of patients with short stature was 1.04 ± 0.11. Mid-parental height (MPH)-SDS in patients with short stature was − 0.57 ± 0.52.

PCDAI scores were assessed in 88 patients with CD whose clinical information was available. They were classified into two groups according to PCDAI scores [inactive/mild (*n* = 41) vs. moderate/severe (*n* = 47)]. Patients in the moderate/severe group (PCDAI score ≥ 30) had lower weight-SDS, BMI-SDS, and IGF-1 SDS than the patients in the inactive/mild group (all *P* < 0.001) (Table 2).

**Table 2:** Clinical and biochemical parameters according to PCDAI scores

	PCDAI < 30 Inactive/mild (*n* = 41)	PCDAI ≥ 30 Moderate/Severe (*n* = 47)	*P* value
Age at diagnosis, years	13.5 ± 1.9	13.6 ± 2.7	0.934
Sex (Male:Female)	32:9	28:19	
PCDAI score	15.85 ± 7.96	45.80 ± 11.23	< 0.001
Short stature	4/41	3/47	0.700
Height-SDS	-0.18 ± 1.08	-0.50 ± 0.97	0.156
Weight-SDS	-0.50 ± 1.17	-1.69 ± 1.32	< 0.001
BMI-SDS	-0.56 ± 1.31	-1.79 ± 1.30	< 0.001
IGF-1 SDS	-0.35 ± 1.30	-1.96 ± 1.17	< 0.001
IGFBP-3 SDS	-1.48 ± 1.31	-4.63 ± 18.41	0.577
BA/CA ratio	0.85 ± 0.11	1.01 ± 0.11	0.034
Delayed puberty	3/41	3/47	1.000
LH, IU/L	2.88 ± 1.28	2.16 ± 1.66	0.104
FSH, IU/L	3.01 ± 1.51	2.12 ± 1.48	0.416
Estradiol, pg/mL (Female)	12.99 ± 3.19	15.21 ± 8.99	0.473
Testosterone, ng/mL (Male)	2.70 ± 2.14	1.32 ± 1.53	0.034
TSH, µIU/mL	2.12 ± 1.11	2.02 ± 1.32	0.784
Free T4, ng/dL	1.28 ± 0.13	1.41 ± 0.20	0.010
25(OH)D_3_, ng/mL	14.23 ± 6.77	15.97 ± 6.52	0.253
Osteoporosis	7/38	9/46	0.813
Lumbar BMD Z-score	-0.55 ± 1.39	-0.84 ± 1.31	0.321

*PCDAI *Pediatric Crohn disease activity index, *IGF-1 *Insulin-like growth factor-1, *IGFBP-3 *Insulin-like growth factor binding protein-3, *BA *Bone age, *CA *Chronological age, *LH *Luteinizing hormone, *FSH *Follicle stimulating hormone, *TSH * Thyroid stimulating hormone, *25(OH)D*_*3*_ 25-hydroxyvitamin D_3_

**Fig. 1:**
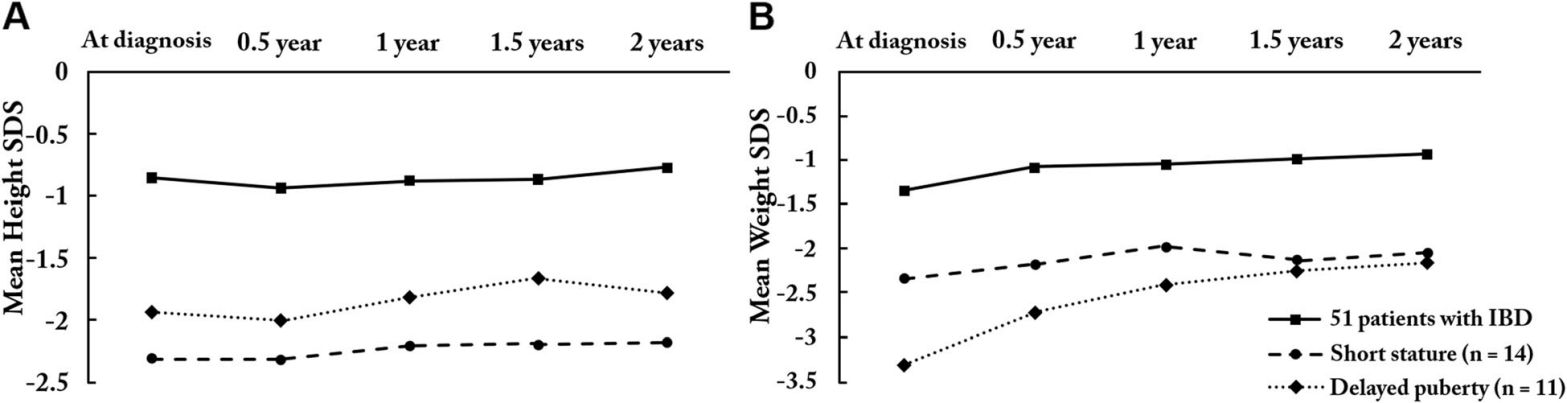
Trends of height-SDS and weight-SDS of patients over two years of treatment **a** Trend of height-SDS **b** Trend of weight-SDS

During a 2-year follow-up period, height-SDS and weight-SDS data were available in 70 patients. Nineteen of 70 patients had a height velocity of less than 2 cm over 2 years. Among the remaining 51 patients, height-SDS did not significantly improved (*P* = 0.958), while weight-SDS was significantly increased (*P* < 0.001) (Fig. 1). The mean age at diagnosis of IBD was 13.5 ± 1.6 years (range, 9.5–15.9 years) in 33 boys and 11.6 ± 3.2 years (1.3–15.5 years) in 18 girls. Weight gain mainly occurred during the first 6 months after treatment. In 14 patients with short stature, significant improvement was not observed in weight-SDS as well as height-SDS.

### Delayed puberty in patients with IBD

Thirty-six girls were above the age of 13 years, and 73 boys were above the age of 14 years at the time of their last follow-up. Among 109 patients (36 girls and 73 boys, 101 CD and 8 UC), 11 patients (6 boys and 5 girls) with CD (10.1 %) had delayed puberty. The mean age at diagnosis of IBD in the 11 patients was 14.3 ± 1.4 years. The mean BA/CA ratio in patients with delayed puberty was 0.84 ± 0.10 (range, 0.73–0.91). Initial height-SDS and weight-SDS at diagnosis of IBD were − 1.94 ± 1.26 and − 3.32 ± 1.90, respectively. The mean initial height- and weight-SDS at diagnosis of IBD in patients without delayed puberty were − 0.28 ± 0.96 and − 1.03 ± 1.20, respectively, which were significantly higher than the height-SDS and weight-SDS of patients with delayed puberty (*P* = 0.001 and 0.001, respectively). The mean BMI-SDS of the patients with delayed puberty was significantly lower than the BMI-SDS of the patients without delayed puberty (-2.77 ± 1.77 vs. -1.15 ± 1.31, *P* = 0.010). Patients in the moderate/severe group (PCDAI score ≥ 30) had significantly lower testosterone concentrations than patients in the inactive/mild group (*P* = 0.034).

Among 5 girls with delayed puberty, one girl manifested menarche at 16 years of age; however, menstruation was no longer observed after her first menstrual period. Another girl showed menarche at the age of 15.5 years. The other 3 girls did not experience menarche by the time of the last follow-up (age, 14.1–15.8 years).

Among 31 patients who showed spontaneous menarche, 8 girls (25.8 %) manifested secondary amenorrhea at diagnosis of IBD at a mean age of 15.1 ± 0.8 years. Serum LH, FSH, and estradiol levels of the 8 girls with secondary amenorrhea were 1.78 ± 0.73 IU/L, 4.49 ± 3.19 IU/L, and 13.59 ± 4.17 pg/mL, respectively.

### Vitamin D deficiency and bone mineral density

Serum 25(OH)D_3_ levels were measured in 115 of 127 patients. The mean 25(OH)D_3_ level was 14.5 ± 6.9 ng/mL (range, 2.2–45.2 ng/mL) (Table 1). Vitamin D deficiency was documented in 81.7 % (94/115) of patients (87 patients with CD [82.1 %, 87/106] and 7 children with UC [77.8 %, 7/9]). Among 94 patients with vitamin D deficiency, 21 patients (22.3 %) had lumbar BMD Z-scores lower than − 2 SDS.

DXA was performed in 119 patients at the time of IBD diagnosis. BMD Z-score was below − 1.0 in 60 of 119 patients (50.4 %). Twenty-five of 119 patients (21.0 %) had BMD Z-scores below − 2.0 (21 patients with CD and 4 patients with UC). In 25 patients with osteoporosis (BMD Z-score ≤ -2.0), the mean BMD Z-score was − 2.34 ± 0.93, and the mean serum 25(OH)D_3_ level was 14.01 ± 5.71 ng/mL (Table 3).

**Table 3:** Clinical and biochemical parameters according to the presence of osteoporosis

	Spine BMD ≤ -2 SDS (*n* = 25)	Spine BMD > -2 SDS (*n* = 94)	*P* value
Sex (male:female)	13:12	66:28	
Age at diagnosis, years	14.3 ± 1.5	13.5 ± 2.1	0.057
PCDAI	36.56 ± 20.16	31.82 ± 17.80	0.410
Delayed puberty	8/25	3/94	< 0.001
Height-SDS at diagnosis	-1.47 ± 1.11	-0.11 ± 0.90	< 0.001
Weight-SDS at diagnosis	-2.40 ± 1.45	-0.99 ± 1.23	< 0.001
BMI-SDS at diagnosis	-2.07 ± 1.36	-1.19 ± 1.35	0.009
Calcium, mg/dL	8.67 ± 0.57	8.94 ± 0.46	0.082
Phosphorus, mg/dL	5.07 ± 0.53	4.28 ± 0.75	0.053
Alkaline phosphatase, IU/L	111.56 ± 38.04	124.65 ± 46.64	0.268
IGF-1 SDS	-1.50 ± 1.46	-1.00 ± 1.21	0.169
IGFBP-3 SDS	-6.64 ± 21.53	-1.21 ± 1.21	0.102
TSH, µIU/mL	2.40 ± 1.25	2.04 ± 1.39	0.222
Free T4, ng/dL	1.37 ± 0.15	1.40 ± 0.20	0.608
LH, IU/L	2.19 ± 2.04	2.71 ± 1.58	0.088
FSH, IU/L	4.18 ± 2.79	2.90 ± 2.04	0.098
Estradiol, pg/mL	17.34 ± 11.35	12.93 ± 3.81	0.657
Testosterone, ng/mL	1.71 ± 1.69	2.54 ± 2.16	0.323
25(OH)D_3_, ng/mL	14.01 ± 5.71	14.26 ± 6.52	0.874
Lumbar BMD Z-score	-2.34 ± 0.93	-0.39 ± 1.07	< 0.001

*BMD *Bone mineral density, *PCDAI *Pediatric Crohn disease activity index, *BMI *Body mass index, *IGF-1 *Insulin-like growth factor-1, *IGFBP-3 *Insulin-like growth factor binding protein-3, *TSH *Thyroid stimulating hormone, *LH *Luteinizing hormone, *FSH *Follicle stimulating hormone, *25(OH)D*_*3*_ 25-hydroxyvitamin D_3_

Height-SDS, weight-SDS, and BMI-SDS at diagnosis of IBD were lower in patients with osteoporosis than in patients without osteoporosis (*P* < 0.001, *P* < 0.001, *P* = 0.009, respectively). Serum 25(OH)D_3_ level positively correlated with lumbar BMD Z-score by Pearson correlation analysis (*r* = 0.215, *P* = 0.024). Lumbar BMD Z-scores were lower in patients with delayed puberty than that in patients without delayed puberty (-2.42 ± 1.30 vs. -0.60 ± 1.23, *P* < 0.001).

Four patients with osteoporosis were treated with pamidronate for 20.0 ± 7.0 months (range, 12–28 months). Three patients who were treated with pamidronate therapy showed their average lumbar BMD Z-scores before and after pamidronate therapy were − 2.93 ± 0.29 and − 1.48 ± 0.56, respectively (*P* = 0.109). Follow-up lumbar BMD in these three patients was measured at 12 to 18 months after pamidronate therapy. In one patient whose lumbar BMD Z-score did not improve after pamidronate therapy, lumbar BMD Z-scores decreased from − 3.64 to -3.76. This patient was diagnosed with CD at the age of 11.4 years, and her height-SDS and weight-SDS at diagnosis of CD were − 1.47 and − 2.62, respectively. Her lumbar BMD was evaluated the age of 13.4, 14.4, and 15.4 years because she did not present pubertal signs until she reached 15.4 years of age. Her lumbar BMD Z-scores were − 2.16, -3.23, and − 3.64 at the age of 13.4, 14.4, and 15.4 years, respectively.

## Discussion

This study evaluated the frequency and outcomes of endocrine complications in children and adolescents with IBD. PCDAI scores were negatively correlated with weight-, BMI-, and IGF-1-SDS, and testosterone level. In addition, low weight-SDS was associated with delayed puberty, and optimal pubertal development was necessary for appropriate BMD in patients with IBD. Thus, clinical severity of IBD is critical to the growth, pubertal development, and bone health of children and adolescents with IBD.

In the present study, 11.0 % of the patients manifested short stature. Growth retardation is more common in CD, because the insidious onset and failure of linear growth are associated with small bowel involvement [22, 46, 47]. Thus, small numbers of patients with UC were referred to our endocrine clinic and enrolled in this study. The prevalence and severity of growth impairment has differed among several previous studies [18, 48]. Growth failure can be described in terms of height-SDS or by variations in growth velocity during 3–4 months [48]. A previous study reported that height Z-scores ≤ -2 SD were seen in 19 % of patients with CD and 5 % of UC patients [49]. However, decreased height velocity was observed in 65 % of CD and 34 % of UC patients [19]. In the present study, 14 out of 127 patients presented with short stature (height Z-scores ≤ -2 SD). We could not investigate height velocity before diagnosis of IBD since this study was a retrospective review of medical records, and some data were not available.

Factors that negatively affect linear growth are increased levels of inflammatory cytokines such as interleukin-6 (IL-6) and tumor necrosis factor-α (TNF-α), malnutrition, suppression of serum IGF-1 levels, and disease severity [11, 50, 51]. Our study showed lower IGF-1-SDSs in patients with higher PCDAI scores (≥ 30), indicating that linear growth was influenced by severity of IBD. Given that growth problems are due to inflammation and undernutrition, catch-up growth after treatment could occur [52]. Our patients did not present significant height-SDS improvement during the follow-up period, while weight-SDS significantly improved. It is thought that significant changes in height-SDS were not shown in patients whose pubertal spurt was attenuated and in cases diagnosed with IBD in late puberty. In the previous study, catch-up growth did not occur due to an attenuated pubertal growth spurt, although they show secondary sexual characteristics within the average pubertal age range [14]. Thus, early diagnosis and intervention are critical for better outcomes from the perspective of growth. Delayed diagnosis of IBD has been found to affect the degree of growth impairment [47, 53, 54].

Inflammation and poor nutrition also affect pubertal development. Our study showed lower testosterone levels in patients with higher PCDAI scores (≥ 30). Increased pro-inflammatory cytokine levels have been associated with reduced serum testosterone levels, which results in delayed puberty [10]. Thus, delayed puberty is more common in patients who have not achieved remission or have active disease [10]. In our study, 10.1 % (11 out of 109) of patients presented with delayed puberty, and they showed significantly low BMI. Another 8 out of 31 who experienced menarche before diagnosis of IBD showed secondary amenorrhea. The relationship between the IBD and menstrual alteration has not been completely explored. Aberration of the pulsatile release of GnRH from the hypothalamus leads to cessation of menstruation, which could occur in response to weight loss [55]. Pubertal delay also contributes to growth retardation. Therefore, early management of disease activity and maintaining remission is essential for optimal growth and pubertal development.

In our study, vitamin D deficiency was documented in 81.7 % (94/115) of patients. Vitamin D deficiency is present in 57–62 % of pediatric IBD patients [56, 57]. Frequency of vitamin D deficiency can be different according to the different cut off level of 25(OH)D_3_ (< 12 ng/mL) [58]. Disease severity can affect vitamin D intake or absorption and bile salt absorption, leading to excessive losses of dietary vitamin D [59]. In addition, it has been suggested that vitamin D deficiency might be a contributing factor in the development of IBD, as vitamin D plays a role in affecting anti-inflammatory adaptive immune function of the gastrointestinal tract [60, 61]. Thus, it is essential to check serum vitamin D levels and supplement vitamin D in patients with IBD.

In the present study, vitamin D levels positively correlated with lumbar spine BMD. There have been some conflicting reports as to whether vitamin D deficiency is a risk factor for low BMD [62, 63]. In a recent study of Asian children, female sex, older age, and low hemoglobin levels further increase risk of low BMD Z-scores rather than vitamin D status in patients with IBD [31]. As for BMD in our study, delayed puberty was more commonly found in patients with osteoporosis, suggesting that timely pubertal development is critical for bone mass accrual. Disease-related chronic inflammation and glucocorticoid treatment are also implicated in impaired bone metabolism. Regarding the recovery of decreased BMD, a recent study showed promising result where bone formation markers such as bone alkaline phosphatase and osteocalcin were increased after treatment using an anti-TNF agent [64]. However, improvement in bone turnover markers has not led to an increase in BMD Z-score [13, 28]. The risk of fracture in pediatric IBD was not higher than age-, sex-, and gender-matched controls in a previous study [65]. In contrast to pediatric patients, adults with IBD are at increased risk of hip fracture, suggesting that regular BMD examination is needed in IBD patients [66].

This study is limited by its reliance on anthropometric data, laboratory findings, and DXA information from medical records, and some records were not available.

## Conclusions

This study demonstrated that vitamin D deficiency and low BMD were the common endocrine complications in children and adolescents with IBD. Clinical severity of IBD is critical to the growth, pubertal development, and bone health of children and adolescents with IBD. Regular assessment of growth parameters, pubertal stage, and BMD is necessary in children and adolescents with IBD for adequate growth and bone health.

## Data Availability

The datasets used and/or analyzed during the current study are available from the corresponding author on reasonable request. This study was approved by the Institutional Review Board of the Asan Medical Center, Seoul, Korea (IRB No. 2017 − 0939). Our study was exempt from the requirement of informed consent because of the retrospective nature of the study and the analysis used anonymous clinical data. Not applicable.

## Abbreviations

BMD: Bone mineral density
IBD: Inflammatory bowel disease
CD: Crohn disease
UC: Ulcerative colitis
25(OH)D_3_: 25-hydroxyvitamin D_3_
SDS: Standard deviation score
BMI: Body mass index
IGF-1: Insulin-like growth factor-1
GH: Growth hormone
PCDAI: Pediatric Crohn disease activity index
IGFBP-3: Insulin-like growth factor binding protein-3
BA: Bone age
LH: Luteinizing hormone
FSH: Follicle stimulating hormone
DXA: Dual energy X-ray absorptiometry
CA: Chronological age
TSH: Thyroid stimulating hormone
IL-6: Interleukin-6
TNF-α: Tumor necrosis factor-α
GnRH: Gonadotropin-releasing hormone
